# First tonic-clonic seizure five hours after Ad26.COV2.S vaccination without acute brain lesion but long-term chorea

**DOI:** 10.1016/j.clinsp.2023.100281

**Published:** 2023-09-07

**Authors:** Antonio-Carlos M. de Almeida, Ana C. Fiorini, Fulvio A. Scorza, Josef Finsterer

**Affiliations:** a Centro de Neurociências e Saúde da Mulher “Professor Geraldo Rodrigues de Lima”, Escola Paulista de Medicina, Universidade Federal de São Paulo (EPM/UNIFESP), São Paulo, SP, Brazil; b Programa de Estudos Pós-Graduado em Fonoaudiologia, Pontifícia Universidade Católica de São Paulo (PUC-SP), São Paulo, SP, Brazil; c Departamento de Fonoaudiologia, Escola Paulista de Medicina, Universidade Federal de São Paulo (EPM/UNIFESP), São Paulo, SP, Brazil; d Disciplina de Neurociência, Universidade Federal de São Paulo, Escola Paulista de Medicina (UNIFESP/EPM), São Paulo, SP, Brazil; e Neurology & Neurophysiology Center, Vienna, Austria

SARS-CoV-2 vaccines of any kind are not free from side effects[1]. They mainly cause neurological side effects affecting both the Central and Peripheral Nervous System (CNS, PNS)[1]. CNS disorders triggered by SARS-CoV-2 vaccines can be complicated by seizures[2]. Seizures can occur without acute structural cerebral damage^3^, ^4^, ^5^. Specifically, seizures are associated with encephalitis[2], Venous Sinus Thrombosis (VST)[6], Acute, Disseminated, Encephalomyelitis (ADEM)[7], and acute, necrotizing encephalitis[8]. Seizures after SARS-CoV-2 vaccination have been reported especially with the brands BNT162b2 (Biontech-Pfizer vaccine [BPV]) and Ad26.COV2.S (Johnson & Johnson vaccine [JJV])[9]. These seizures occurred within a few days after vaccination. Seizures within hours of JJV application have not been reported.

The patient is a 32-year-old female, height 165 cm weight 48 kg, who presented after a first tonic-clonic seizure five hours after JJV. She had neither a tongue bite nor urinary leakage. After a reorientation phase of 2 min, she regained her preictal state but noticed severe headaches up to VAS-9. Blood tests only revealed mild hyper-CKemia. Cerebral computed tomography, CT-angiography, and CT-venography were normal. Cerebral Magnetic Resonance Imaging (MRI) the next day showed sparse, T2-hyperintense, punctate subcortical lesions with a maximum diameter of 4 mm. The electroencephalogram was inconclusive. Structural epilepsy was diagnosed and treatment with lacosamide (200 mg/d) started. The headache disappeared after taking nonsteroidal, anti-inflammatory drugs.

The history was positive for polydrug-mania (speed, LSD, cocaine) for six months until age 13 when she went into cardiopulmonary arrest after sniffing a suspected cocaine/heroin mixture. Although she was successfully resuscitated by the Cardio-Pulmonary Resuscitation (CPR) rescue team, upon awakening from the coma, she developed left predominant quadrispasticity along with choreatiform hyperkinesias, that increased in intensity with exertion. Despite her impairment, the patient gave birth to three healthy children and was able to adequately care for them. As a result of the spasticity, she developed scoliosis, hyperlordosis, and pelvic tilt. She received a single dose of BPV four months after the JJV with no complications.

Neurologic examination revealed mild dysarthria, repeated dyskinesias of facial muscles, mild bilateral weakness for finger-spreading (M5-), bilateral dysdiadochokinesia, left finger-nose passing, increased tendon reflexes on upper and lower limbs, choreatiform movements with left-sided predominance, unfixed flexion contractures of hands and fingers, unfixed inversion of the feet, spastic gait with a tendency to fall, pelvic tilt and lumbar hyperlordosis. A recent cerebral MRI was unchanged to the previous one (Fig. 1).

**Fig. 1:**
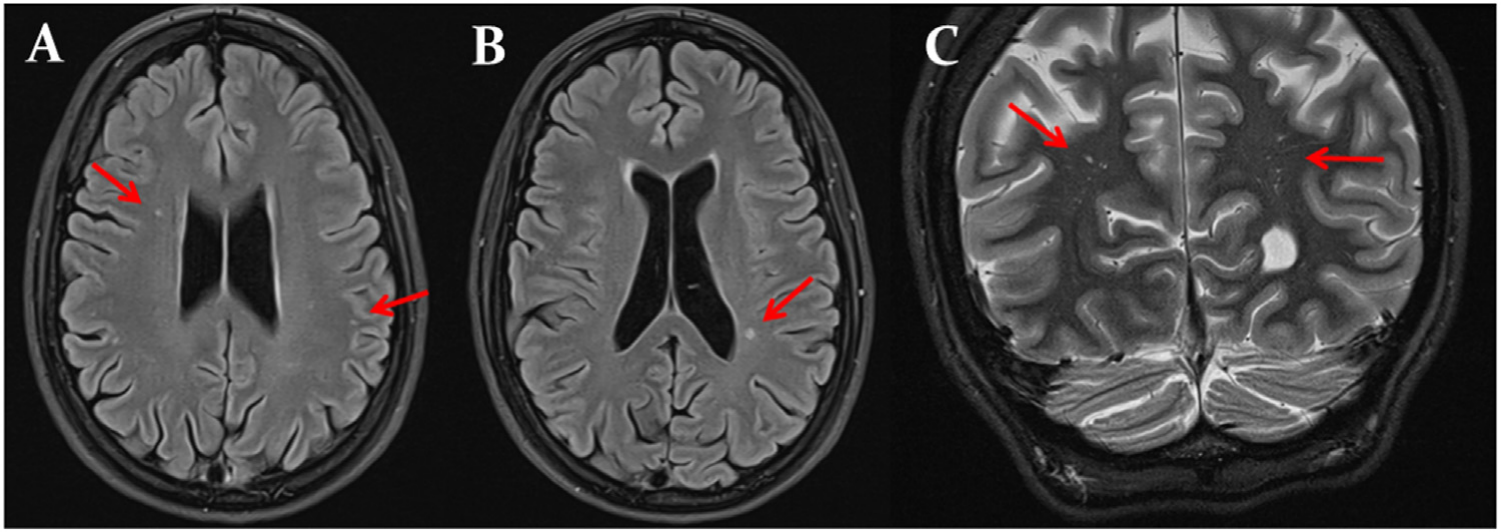
Multimodal cerebral MRI at the age of 32 shows mild global atrophy and multiple T2-yperintense spots within the white matter bilaterally (panels A, B, and C).

A trial of a single dose of sulpiride was discontinued by the patient because of lactorrhoea. She is currently taking lacosamide, tiapride, and escitalopram with beneficial effects.

The index patient is interesting due to a first tonic-clonic seizure five hours after a JJV dose. Although seizures have been reported as a complication of JJV[9], these seizures occurred with a latency period of days after vaccination. Seizures within <24 h after vaccination have not been reported. The pathophysiological background of the seizure remains speculative. She had never had seizures in her life before, including after CPR at age 13 or during the multi-drug episode. Her family history was negative for epilepsy. She suffered no traumatic brain injury, encephalitis/meningitis, and did not drink alcohol or use illegal drugs after the age of 13. There was no sleep deprivation or acute infection to explain the seizure.

Since no plausible cause of the seizure could be determined, it was traced back to the JJV. The mechanism by which JJV triggered the seizure remains speculative. There was no encephalitis, VST, ADEM, or cerebral bleeding triggered by the vaccination that could explain the seizure. Whether the previous damage from hypoxia at the age of 13 contributed to the onset of the seizure, remains speculative but is plausible. It has been shown that pre-existing structural damage to the brain generally favors the development of cerebral side effects after a SARS-CoV-2 vaccination[10]. It is also conceivable that the patient experienced a transient VST, that was complicated by the seizure but resolved spontaneously due to autoanticoagulation. An argument for transient VST could be the severe headache she experienced shortly after the seizure. It is also plausible that activation of the immune system or electrolyte imbalances led to the destabilization of the cortical neuronal membranes and thus to the seizure. However, most parameters in the serum were normal.

Overall, this case demonstrates that JJV can induce generalized seizures, repeat vaccination with a different brand after four months is safe and does not induce seizure recurrence, and prior brain damage may favor the development of seizures after vaccination.

## Declarations

Ethics approval: Was in accordance with ethical guidelines. The study was approved by the institutional review board.

Consent to participate: Was obtained from the patient.

Consent for publication: Was obtained from the patient.

Availability of data: All data are available from the corresponding author.

Code availability: Not applicable.

## Funding

No funding was received.

## Declaration of Competing Interest

The authors declare no conflicts of interest.
